# The Relationship Between Cognitive Dysfunction Through THINC-Integrated Tool (THINC-it) and Psychosocial Function in Chinese Patients With Major Depressive Disorder

**DOI:** 10.3389/fpsyt.2021.763603

**Published:** 2021-11-23

**Authors:** Han Han, Yanyan Hou, Shuqiao Yao, Shaohua Hu, Qi Zhou, Xin Yu, Roger S. McIntyre, Chuan Shi

**Affiliations:** ^1^Beijing Huilongguan Hospital, Peking University Huilongguan Clinical Medical School, Beijing, China; ^2^Peking University Sixth Hospital, Peking University Institute of Mental Health, Beijing, China; ^3^National Health Commission (NHC) Key Laboratory of Mental Health (Peking University), National Clinical Research Center for Mental Health Disorders (Peking University Sixth Hospital), Beijing, China; ^4^Qingdao Mental Health Center, Qingdao University, Shandong, China; ^5^Medical Psychological Center, The Second Xiangya Hospital Central South University, Changsha, China; ^6^Department of Psychiatry, First Affiliated Hospital, Zhejiang University School of Medicine, Hangzhou, China; ^7^The Key Laboratory of Mental Disorder's Management of Zhejiang Province, Hangzhou, China; ^8^Department of Psychiatry, University of Toronto, Toronto, ON, Canada; ^9^Mood Disorders Psychopharmacology Unit, Brain and Cognition Discovery Foundation (BCDF), Toronto, ON, Canada; ^10^Depression and Bipolar Support Alliance (DBSA), Chicago, IL, United States; ^11^Academician workstation of Mood and Brain Sciences, Guangzhou Medical University, Guangzhou, China; ^12^College of Medicine, Korea University, Seoul, South Korea; ^13^Department of Psychiatry and Neuroscience, School of Medicine, University of California, Riverside, Riverside, CA, United States

**Keywords:** MDD, cognitive function, psychosocial function, work productivity, THINC-it

## Abstract

**Background:** Herein, we validate the psychometric properties of the Chinese version of the THINC-integrated tool (THINC-it) as a screening tool for cognitive deficits in patients with major depressive disorder. The primary aim of this study is to determine whether cognitive deficits as detected by the THINC-it tool in adults with major depressive disorder (MDD) are associated with workplace productivity and/or psychosocial function.

**Methods:** Subjects aged 18–65 (*n* = 91) with MDD were evaluated and compared to age-, sex- and education- matched healthy controls (*n* = 95). Symptoms of cognitive dysfunction, workplace productivity, and psychosocial function were measured using the THINC-it tool, Hamilton Depression Scale (HAMD), Sheehan Disability Scale (SDS), The Work Productivity and Activity Impairment questionnaire- Specific Health Problem (WPAI-SHP).

**Results:** There were significant differences in THINC-it scores (*p* < 0.01), the average of HAMD total score (*p* < 0.01) and all aspects of SDS (*p* < 0.01) between two groups. There were significant differences in the four aspects of WPAI between the two groups in the employed status (*p* <0.01). THINC-it subjective cognition and SDS total score, SDS work/school, SDS social, SDS family showed significantly correlation (*r* ranging from 0.255 to 0.386, *p* <0.01). SDS and THINC-it Objective cognition, THINC-it comprehensive cognition were no correlation between two groups. HAMD total score and SDS total score, SDS social, SDS family showed significantly correlation (*r* ranging

## Introduction

Major depressive disorder (MDD) is common, debilitating and associated with significant human capital costs (1, 2). The majority of patients with MDD do not regain premorbid levels of psychosocial functioning despite the resolution of core depressive symptoms. A priority research vista is to identify mediators of functional outcome in persons with MDD (3). Evidence indicates that cognitive functions play a critical role in mediating functional outcomes in MDD (4, 5).

Subjective and objective cognitive functions are not correlate. Moreover, evidence indicates that Perceived Deficits Questionnaire (PDQ) scores correlate with patient reported outcomes (6). According to a study assessing cognitive and social dysfunction in European and Asian populations, the severity of MDD was the main predictor of dysfunction, and impairment of subjective cognitive function was an independent and important predictor of dysfunction. Moreover, only the subjective five-item Perception Deficits Questionnaire (PDQ-5-D) was used to assess the cognitive dysfunction (7–9).

There are insufficient studies evaluating the mediational role of cognitive deficits in persons affected by MDD in China. Our team has previously validated the psychometric properties of the Chinese version of the THINC-integrated tool (THINC-it) for cognitive symptoms in patients with MDD. The Chinese version of THINC-it was a sensitive cognitive assessment tool and can be easily applied to clinical practice (10, 11). The overarching aim of the study herein is to comprehensively evaluate the cognitive function, psychosocial function and work productivity in patients with MDD with an aim to identify a correlation between depression severity, cognitive impairment and psychosocial dysfunction. Our initiative was an attempt to extend previous work documenting the association between THINC-it-measured cognitive deficits in MDD and psychosocial dysfunction (12).

## Methods

This was a multi-center, cross-sectional study. The protocol for this study was approved by the Ethics Committee of Peking University Sixth Hospital. After receiving description of the aims of this study, participants provided written informed consent prior to enrollment. Firstly, we should collect the basic information of the participants. Next, participants were assessed clinical symptoms (HAMD-17), cognitive function (the Chinese version of THINC-it), psychosocial function (SDS) and Workplace Function (WPAI-SHP) by professionally trained psychiatrist.

### Sample

Patients with MDD were recruited from the psychiatric outpatients in three hospitals (Peking University Sixth Hospital, the Second Xiangya Hospital of Central South University, and the First Affiliated Hospital of Zhejiang University) from May 2018 and February 2019. To facilitate sampling and comparison, healthy controls were recruited from the corresponding community of each hospital by the head of each center via advertisement or introduction (Peony Garden Community in Haidian District, Beijing; Furong District, Changsha City, Hunan Province; Qingchun Road District, Hangzhou City). The patients enrolled were matched with healthy controls in age, gender and education. A meta-analysis revealed significant moderate cognitive dysfunction in patients with MDD relative to controls (Cohen's d effect sizes ranging from −0.34 to −0.65). The sample size will be calculated using G ^*^ Power Software (13), we planned to recruit 100 patients with MDD and 100 healthy controls. Actually, a total of 241 subjects were recruited, including 117 patients with MDD and 124 healthy controls.

The criteria for the enrollment of patients with MDD: (1) patients aged 18–65 years who received treatment as outpatients; (2) patients diagnosed with MDD according to the ICD-10 diagnostic criteria; (3) patients with a Hamilton Depression Scale (HAMD) score ≥17; (4) The current depressive episode lasting at least 1 month; (5) patients with a Wechsler Abbreviated Scale of Intelligence (WAIS) score >80; (6) patients provided informed written consent. Exclusion criteria: (1) patients have or have had any of the following diagnoses: alcohol and/or substance use disorder; autism spectrum disorder; bipolar disorder; cerebral palsy; inflammatory demyelinating diseases of the Central Nervous System; brain tumors; dementia, Parkinson's disease or any other degenerative diseases of the Central Nervous System; learning disabilities; Schizophrenia or other mental disorders; other medical conditions that may affect cognitive function (such as: brain tumors, multiple sclerosis, etc.); (2) patients who were taking any drugs that might affect cognitive function, such as glucocorticoids, β-blockers, opioid analgesics, central stimulants, etc.; (3) Patients who took benzodiazepines within 12 h before the THINC-it test. (4) patients who consumed alcohol within 8 h before the THINC-it test; (5) patients who has received electroconvulsive treatment (ECT) within the past 6 months; (6) Patients who could not read or understand informed consent or self-report questionnaires.

The criteria for the enrollment of healthy controls: (1) healthy controls aged 18–65 years; (2) healthy controls with a Wechsler Abbreviated Scale of Intelligence (WAIS) score >80; (3) healthy controls has signed the informed consent. Exclusion criteria: (1) healthy controls has or has had any of the following diagnoses: alcohol and/or substance use disorder; autism spectrum disorder; mood disorder; dementia or any other neurodegenerative diseases; learning disabilities; Schizophrenia or other mental disorders; other medical conditions that may affect cognitive function (e.g., cerebral palsy; inflammatory demyelinating diseases of the Central Nervous System; brain tumors; dementia, Parkinson's disease or any other degenerative diseases of the Central Nervous System etc.); (2) healthy controls have unstable medical diseases; (3) healthy controls who were taking any drugs that might affect cognitive function such as glucocorticoids, β-blockers, opioid analgesics, central excitement Agents, etc.; (4) healthy controls who consumed alcohol within 8 h before the THINC-it test; (5) healthy controls who could not read or understand informed consent or self-report questionnaires.

### Data and Instruments

In our study, two psychiatrists with extensive clinical experience administered the HAMD-17 to assess depression. Other measurement tools (THINC-it, SDS, WPAI-SHP) were implemented by a professionally trained psychiatrist. All participants were tested in a quiet room. THINC-it was administered on desktop computers and touchscreen tablet devices, and other tools were administered on paper. We have described in more detail in the following sections.

The severity of depression was assessed by the 17-item HAMD (HAMD-17), and divided into: total score ≥24 for severe depression, ≥17 for moderate depression, and ≤ 7 for without depression symptoms (14, 15).

Cognitive assessment: A simplified Chinese version of THINC-it Set was used for cognitive assessment. THINC-it contained subjective cognitive test (PDQ-5) and 4 objective neuropsychological tests (Spotter, SPO; Symbol Check, SC; Code Breaker, CB; Trails, TMT-B), which could measure multiple cognitive dysfunction areas (i.e., attention, working memory, information processing speed and executive function, etc.) of patients with MDD. The English and Chinese versions of the THINC-it tool showed that the tool had high reliability and validity and could screen for cognitive dysfunction effectively (10, 11).

Psychosocial function: Sheehan Disability Scale (SDS) was used to assess the psychosocial function in patients with MDD (16). SDS was a self-rating scale, which includes three aspects of psychosocial function, work/school, social function and family function/family responsibility. Additionally, the scale can measure the days lost due to symptoms (in the last 7 days) and days that were unproductive (in the last 7 days) (17, 18). Each subscale scores ranged from 0 (unimpaired) to 10 (highly impaired), and total function impairment scores was computed ranging from 0 (unimpaired) to 30 (highly impaired). A higher SDS score indicates greater functional impairment.

Workplace Function: The Work Productivity and Activity Impairment questionnaire-Specific Health Problem (WPAI-SHP) is a 6-item self-rating scale that provides quantitative measure of work efficiency and activity impairment in the last 7 days due to specific health problem (19). The subjects were required to fill in the specific time of miss work or actually work, and select effect degree in the work and daily activities. It was divided into four indicators for evaluation, including absenteeism (percent work time missed), presenteeism (percent time impaired while working), overall work impairment (percent overall work impairment), and overall activity impairment. Higher scores indicated worse work productivity or activity impairment.

Intelligence assessment: The third edition of the WAIS was performed to obtain an intelligence quotient. The intelligence quotient consisted mainly of four speech scale scores, including common sense, arithmetic, similarity, and digital breadth.

### Statistical Analysis

Statistical analyses excluded individuals with incomplete data on the THINC-it, SDS and WPAI scale. SPSS22.0 was used for statistical analysis. In the descriptive analysis of the subjects, the continuous numerical variables were expressed as mean (standard deviation), and the categorical variable was expressed by frequency (percentage). In the observation index analysis, independent sample *t*-test was used to continuous numerical variables between MDD and healthy controls (HC), and the chi-square test was used to categorical variable.

Cognitive function and scale scores: *Z*-scores were created for all continuous measures (THINC-it, HAMD, SDS, WPAI), and the formula was [participant score on test – mean of HC on test] ÷ standard deviation of HC on test *Z*-scores were adjusted in direction, and higher *Z*-score indicated better performance. The subjective cognitive test score was the PDQ-5 score, Objective cognitive test composite score was the sum of four objective cognitive test *Z*-scores, and the total comprehensive cognitive score was the total of the 5 sub-test *Z*-scores. Calculate *Z*-score's mean (standard deviation) of cognitive function and scales, and independent sample *t*-test was used to compare the differences between MDD and HC.

The Pearson correlation was used to calculate the correlation between cognitive function (i.e., as measured by the THINC-it), degree of depression (i.e., as measured by the HAMD), and social function (i.e., as measured by the SDS) in persons with MDD and HCs. Employed people were selected from the patients with MDD for subgroup analysis, and the Pearson correlation was used to compare the correlation between cognitive function (i.e., THINC-it scores) and work efficiency and daily activity ability (i.e., WPAI scores). Lastly, the analysis of variance was used to estimate the difference in cognitive function between the employed and unemployed in patients with MDD.

## Results

### Demographic Information

Overall, 91 patients with MDD and 95 healthy controls completed the THINC-it, HAMD, SDS, and WPAI in our study. Significant differences were found in education level (*t* = −3.45, *p* < 0.01) and IQ scores (*t* = −5.16, *p* < 0.01) between HC and persons with MDD. The education level and IQ of patients with MDD were lower than HC; there was no difference between the two groups in terms of age, gender, and the employment status. Significant differences in the four aspects of WPAI (i.e., absenteeism, presenteeism, overall work impairment and overall activity impairment) were observed between the two groups in the employed status (*p* < 0.01) (Table 1).

**Table 1:** Demographic information between MDD and HC.

**Item**	**MDD, *n* = 91**	**HC, *n* = 95**	**T/χ^2^**	***p*-value**
	**M ± SD/*n* (%)**	**M ± SD/*n* (%)**		
Age (year)	29.57 ± 10.97	27.09 ± 7.57	1.79	0.076
Education level (year)	14.27 ± 3.18	15.76 ± 2.65	−3.45	<0.01
IQ scores	117.09 ± 16.55	130.27 ± 18.23	−5.16	<0.01
Sex				
Male	35 (38.5%)	48 (50.5%)		0.98
Female	56 (61.5%)	47 (49.5%)		
Employed (%)	36 (39.6)	40 (42.1%)		0.724
Employed (%)				
WPAI absenteeism	36.97 ± 37.79	39.62 ± 24.08		<0.01
WPAI presenteeism	39.62 ± 24.08	0.00 ± 0.00		<0.01
WPAI overall work impair	60.67 ± 31.59	0.00 ± 0.00		<0.01
WPAI overall activity impair	38.33 ± 27.31	0.00 ± 0.00		<0.01

### Differences Between the Two Groups

Compared with HC, the average HAMD total score and THINC-it scores were significantly higher in the MDD group (*p* < 0.01). In terms of psychosocial function, the average SDS total score and three subscale scores were significantly higher in patients with MDD (*p* < 0.01) (Table 2). Also, the days lost due to symptoms and the days unproductive in patients with MDD increased significantly during the past week (*p* < 0.01) (Table 2).

**Table 2:** Comparison of depression degree, cognitive function and social function.

	**MDD, *n* = 91**	**HC, *n* = 95**	**T/χ2**	***p*-value**
	**M ± SD**	**M ± SD**		
HAMD total	22.87 ± 4.43	0.33 ± 1.09	47.2	<0.01
SDS total	13.48 ± 7.21	0.12 ± 0.82	17.59	<0.01
SDS- work/school	4.81 ± 2.68	0.03 ± 0.31	16.9	<0.01
SDS -social	4.41 ± 2.69	0.04 ± 0.29	15.41	<0.01
SDS-family	4.26 ± 2.72	0.04 ± 0.29	14.75	<0.01
the days lost	2.75 ± 2.64	0	9.94	<0.01
The days unproductive	4.27 ± 2.69	0.04 ± 0.32	14.92	<0.01
THINC-it objective cognition (*Z* score)	−0.40 ± 0.92	0.00 ± 0.74	−3.22	<0.01
THINC-it subjective cognition (*Z* score)	−2.39 ± 1.61	0.00 ± 1.00	−12.12	<0.01
THINC-it comprehensive cognition (*Z* score)	−0.80 ± 0.77	0.00 ± 0.60	−7.86	<0.01

### Correlation Analysis in MDD

In MDD, THINC-it subjective cognition and SDS total score (*r* = 0.352, *p* < 0.01), SDS work/school (*r* = 0.386, *p* < 0.01), SDS social (*r* = 0.255, *p* < 0.01), SDS family (*r* = 0.299, *p* < 0.01) were positively correlated. There was no correlation between THINC-it Objective cognition and SDS. There was no correlation between THINC-it comprehensive cognition and SDS. HAMD total score and SDS total score (*r* = 0.220, *p* < 0.01), SDS social (*r* = 0.219, *p* < 0.01), SDS family (*r* = 0.208, *p* < 0.01) were positively correlated (Table 3).

**Table 3:** Correlation analysis in the degree of depression, cognitive function and social impairment.

**Item**	**THINC-it subjective cognition**	**THINC-it objective cognition**	**THINK-it comprehensive cognition**	**HAMD total score**	**SDS work/school**	**SDS social**	**SDS family**	**SDS score**
THINC-it subjective cognition	1							
THINC-it objective cognition	−0.121	1						
THINC-it comprehensive cognition	0.302^**^	0.909^**^	1					
HAMD total score	−0.018	0.022	0.013	1				
SDS work/school	0.386^**^	−0.161	0.007	0.160	1			
SDS social	0.255^*^	−0.101	0.010	0.219^*^	0.707^**^	1		
SDS family	0.299^**^	−0.119	0.011	0.208^*^	0.650^**^	0.716^**^	1	
SDS score	0.352^**^	−0.142	0.011	0.220^*^	0.881^**^	0.906^**^	0.886^**^	1

* *p < 0.05*.

** *p < 0.01*.

### Subgroup Analysis

In MDD group, there were 36 employed and 55 unemployed patients. There were no differences in education, HAMD scores and IQ scores except for age (*p* < 0.01) and gender (*p* = 0.01) between the employed and unemployed patients. The differences in THINC-it test between the analysis groups were compared, age and gender were included in the model for covariance analysis. The result showed that there were no significant differences in all aspect of THINC-it scores (Table 4).

**Table 4:** General information between employed and unemployed in MDD.

	**Employed, *n* = 36**	**Unemployed, *n* = 55**	**T/χ2**	***P***
	**M ± SD/*n* (%)**	**M ± SD/*n* (%)**		
Age (year)	33.69 ± 9.58	26.87 ± 11.06	3.30	<0.01
Education level (year)	15.00 ± 2.80	13.80 ± 3.34	1.78	0.078
HAMD scores	22.19 ± 4.11	23.31 ± 4.61	−1.176	0.243
IQ scores	115.81 ± 17.70	117.93 ± 15.86	−0.596	0.553
Sex				
Male	8 (22.2%)	27 (49.1%)		0.010
Female	28 (77.8%)	47 (50.9%)		
SPO	−0.70 ± 1.12	−0.34 ± 1.29		0.596
SC	−0.35 ± 0.64	0.05 ± 1.07		0.646
CB	−0.48 ± 0.75	−0.60 ± 1.21		0.157
TMT-B	−0.67 ± 1.93	−0.28 ± 1.61		0.715
THINC-it objective cognition (*Z* score)	−0.55 ± 0.80	−0.29 ± 0.99		0.780
THINC-it subjective cognition (Z score)	−2.05 ± 1.82	−2.62 ± 1.43		0.130
THINC-it comprehensive cognition (*Z* score)	−0.85 ± 0.73	−0.76 ± 0.80		0.333

### Correlation Analysis in Subgroup

Pearson correlation coefficient was used to evaluate the correlation between cognitive function and work productivity in patients with MDD, and the results showed that THINC-it subjective cognition was significantly positively correlated with the presenteeism (*r* = 0.468, *p* < 0.05), work productivity loss (*r* = 0.466, *p* < 0.05) and overall activity impairment (*r* = 0.394, *p* < 0.05) of WPAI. There was no correlation between THINC-it Objective cognition and all subdomains of WPAI (*p* > 0.05) (Table 5).

**Table 5:** Correlation analysis between THINC-it and WPAI.

**Item**	**WPAI absenteeism**	**WPAI presenteeism**	**WPAI overall work impairment**	**WPAI overall activity impairment**	**THINC-it Subjective cognition**	**THINC-it Objective cognition**	**THINC-it comprehensive cognition**
WPAI absenteeism	1						
WPAI presenteeism	0.276	1					
WPAI overall work impairment	0.840^**^	0.817^**^	1				
WPAI overall activity impairment	0.408^*^	0.703^**^	0.628^**^	1			
THINC-it subjective cognition	0.327	0.468^*^	0.466^*^	0.394^*^	1		
THINC-it objective cognition	−0.227	−0.017	−0.208	−0.166	−0.037	1	
THINC-it comprehensive cognition	−0.031	0.197	0.057	0.050	0.468^**^	0.866^**^	1

* *p < 0.05*.

** *p < 0.01*.

## Discussion

This is the first study conducted in China that replicates the validation of the THINC-it as a sensitive tool to detect cognitive deficits in adults with MDD. We extend knowledge further, herein, by also determining that subjective deficit in cognitive function highly correlate with disparate measures of psychosocial function.

Cognitive impairment is one of the main manifestations of MDD (20–22). The THINC-it tool is a simple cognitive screening tool, divided into subjective cognition and objective cognition assessment, including attention, information processing speed, executive function, memory and learning function, which can comprehensively measure cognitive impairment in MDD. The THINC-it has been validated at home and abroad (10, 11, 23). Our team has previously validated and reported on psychometric characteristics of the Chinese version of the THINC-it tool, and it is also the first-ever clinical cognitive screening tool applied to Chinese patients with MDD (11). In the study herein, through the THINC-it test, the cognitive function including subjective cognition, objective cognition and comprehensive score of MDD were significantly different from healthy controls. In accordance with previous research, the cognitive function of patients with MDD was significantly impaired when compared to healthy individuals (10, 13, 23, 24).

The study herein reports that social function is significantly impaired in patients with MDD in accordance with previous studies (5, 6, 9, 12). Our findings also indicate that work/school function was more significantly impaired than other subdomains measured by the SDS in persons with MDD. The foregoing finding is also in accordance with what has been reported in Western populations (6). We also identified that work productivity was significantly impaired. And absenteeism was similar to presenteeism in patients with MDD, which was different with the previous studies. In Western samples, presenteeism has disproportionately accounted for impairment in workplace function relative to absenteeism (6, 25). We hypothesize that differences between Western and Asian workplace environments may account for the differential effects of absenteeism and presenteeism on overall workplace impairment.

We also found that subjective cognitive impairment was significantly positively correlated with psychosocial dysfunction (SDS) (*p* < 0.01), however, the overall correlation coefficient was relatively low. Moreover, there was no correlation between objective cognitive impairment and psychosocial dysfunction, which is also confirmed in many previous researches (6, 8, 9, 12, 25). Subjective cognition was highly correlated with depressive symptoms, to a much greater extent than objective cognitive functions. We also found that work/school function was not correlated with depression severity. It is hypothesized that cognitive impairment may have greater contribution to work/school function. Moreover, our study results are in accordance with the foregoing hypothesis as we observed a stronger correlation between subjective cognitive impairment and work/school than other domains of functional impairment in adults with MDD.

Baune, Miller et al. and Clark, DiBenedetti et al.'s studies reported that cognitive dysfunction may play an important role in the relationship between employment and depression (26, 27). We observed a positive correlation between cognitive function and social function in our study, indicating that the impact of cognitive function on social function was an independent contributing factor (7). Herein, we found no correlation between the severity of depression and cognitive impairment. Our conclusion is in accordance with previous studies (26). However, we also observed cognitive impairment persisted despite improvement of depressive symptoms, suggesting that cognitive dysfunction in MDD may be independent of other MDD symptoms (28).

Herein, we observed that presenteeism, overall work impairment and activity impairment were positively correlated with subjective cognition in MDD patients. Both objective and subjective cognitions were not correlated with absenteeism, which is in accordance with previous studies (6, 25). A possible explanation is that the absence rate may not be influenced by cognitive dysfunction, and related to other aspects of absenteeism (e.g., obstacles in asking for work leave such as stigma and discrimination with respect to mental illness) (29). However, cognitive function is associated with a reduction in work efficiency, which may also affect absenteeism (29, 30).

Through subgroup analysis, it was found that there is no significant difference in cognitive function between employed and unemployed in MDD, which is not in accordance with studies published in foreign populations (21, 26, 29). For example, Baune et al.'s study reported that employed patients exhibited significantly greater deficits in a variety of cognitive measures (e.g., language and delayed memory) when compared to employed individuals.

## Conclusion

Herein, we demonstrate that the THINC-it tool is easy to implement, brief in administration and capable of detecting clinically meaningful cognitive deficits in persons with MDD.

The strengths of our study are the representative of the patients that were enrolled, the use of standardized and validated clinical metrics, and the emphasis on both subjective and cognitive functions.

The limitations of our study are that we did not adjust for multiple covariates that may have confounded study findings (e.g., concomitant medications, comorbidities). Moreover, our sample was from some regions of China that may not generalize to all persons in China living with depression. Future studies should determine whether improving cognitive functions as measured by the THINC-it tool reduce psychosocial impairment and workplace disability.

## Data Availability Statement

The original contributions presented in the study are included in the article/supplementary material, further inquiries can be directed to the corresponding author/s.

## Ethics Statement

The studies involving human participants were reviewed and approved by the Medical Ethics Committee of Peking University Sixth Hospital. The patients/participants provided their written informed consent to participate in this study.

## Author Contributions

HH carried out the studies, collecting data, statistical analysis, and drafted the manuscript. YH carried out the studies, data collection, and statistical analysis. SY, SH, and QZ carried out the studies and data collection. XY participated in data collection, analysis, and interpretation. RM participated in the analysis and interpretation of data and drafted the manuscript. CS contributed to the study's design, statistical analysis, data interpretation, manuscript draft, and article review. All authors have read and approved the final manuscript.

## Funding

This research was funded by Chinese Neuropsychological Normative Project (CN-NORM) among the middle-aged and elderly and Lundbeck (Beijing) Pharmaceutical Consulting Co, Ltd. (No. 17746T). Lundbeck (Beijing) Pharmaceutical Consulting Co, Ltd. funder was not involved in the study design, collection, analysis, interpretation of data, the writing of this article or the decision to submit it for publication.

## Conflict of Interest

CS was employed by the company Lundbeck and received honoraria from Lundbeck, Eli Lilly, Janssen, Pfizer, GlaxoSmithKline, and Sanofi- Aventis; industry research grants from Lundbeck; and research grant from the National Key R&D Program of China (2018YFC1314200); and participate in advisory boards for Lundbeck and Sanofi-Aventis. RM has received research grant support from CIHR/GACD/Chinese National Natural Research Foundation; speaker/consultation fees from Lundbeck, Janssen, Purdue, Pfizer, Otsuka, Takeda, Neurocrine, Sunovion, Bausch Health, Novo Nordisk, Kris, Sanofi, Eisai, Intra-Cellular, NewBridge Pharmaceuticals, Abbvie. RM is a CEO of Braxia Scientific Corp. The remaining authors declare that the research was conducted in the absence of any commercial or financial relationships that could be construed as a potential conflict of interest.

## Publisher's Note

All claims expressed in this article are solely those of the authors and do not necessarily represent those of their affiliated organizations, or those of the publisher, the editors and the reviewers. Any product that may be evaluated in this article, or claim that may be made by its manufacturer, is not guaranteed or endorsed by the publisher.
